# Clinical characteristics of thrombospondin type-1 domain-containing 7A-associated membranous nephropathy

**DOI:** 10.1080/0886022X.2020.1819318

**Published:** 2020-09-16

**Authors:** Tomo Suzuki, Wei Han, Shiika Watanabe, Maho Terashita, Mayumi Nakata, Daisuke Ichikawa, Sayuri Shirai, Yugo Shibagaki

**Affiliations:** a Division of Nephrology and Hypertension, Department of Internal Medicine, St. Marianna University School of Medicine, Kanagawa, Japan; b Department of Nephrology, Kameda Medical Center, Chiba, Japan; c Division of Nephrology and Hypertension, Department of Internal Medicine, St. Marianna University School of Medicine Yokohama City Seibu Hospital, Yokohama, Japan

Membranous nephropathy (MN) is a common cause of nephrotic syndrome. Phospholipase A2 receptor 1 (PLA2R) and thrombospondin type-1 domain-containing 7 A (THSD7A) present in the podocyte membrane are the major antigens causing primary MN. The prevalence of THSD7A-associated MN (THSD7A-MN) is low, around 10% among primary MN cases reported from various countries [1,2]. Furthermore, THSD7A-MN is often associated with malignancies [3]. However, other clinical features of THSD7A-MN are still unclear. Here, we performed a single-center cohort study of primary MN and studied PLA2R and THSD7A antibody expression by immunofluorescence staining. We also compared the clinical characteristics between THSD7A-MN and PLA2R-MN patients. This study was approved by the Institutional Committee on Human Research of our institution (approval No. 4506).

The subjects were 22 IgG4-dominant, primary MN patients. The patients underwent kidney biopsy without an apparent secondary cause. We performed immunofluorescence (IF) staining for determining PLA2R and THSD7A levels using frozen biopsy samples. Thereafter, we tried to search for potential concomitant existence of malignancy by computed tomography, fiber gastroscopy, and colonoscopy.

If staining results revealed that 3 of 22 patients (13.6%) were negative for both PLA2R and THSD7A, nobody was positive for both, leaving 19 patients (14 with PLA2R, 5 with THSD7A).

We compared the baseline and clinical characteristics of PLA2R-MN and THSD7A-MN (Table 1). Male patients accounted for less in those with THSD7A-MN (THSD7A group) than in those with PLA2R-MN (PLA2R group), although this difference did not reach statistical significance (20 vs. 57.1%, *p* = 0.15). No difference was found in terms of age and renal function. However, THSD7A-MN group had significantly higher urinary protein levels [THSD7AMN vs. PLA2R-MN, 7900 (5327, 8778) vs. 2647 (1114, 4980) g/gCre, *p* = 0.049], significantly lower serum IgG levels [540 (271,623) vs. 654 (593, 1033) mg/dL, *p* = 0.02], and total protein levels [4.6 (3.6, 4.7) vs. 5.5 (4.8, 6.3) g/dL, *p* = 0.08]. Regarding comorbidities, malignancy was found relatively more in THSD7A-MN group than in PLA2R-MN group (40 vs. 7.1%, *p* = 0.08). Allergic disease was significantly more prevalent in THSD7A-MN group than in PLA2R-MN group (60 vs. 7.1%, *p* = 0.01).

**Table 1.** Comparison of phospholipase A2 receptor 1 (PLA2R) and thrombospondin type-1 domain-containing 7 A (THSD7A) characteristics in patients with membranous nephropathy.

	THSD7A-MN (*N* = 5)	PLA2R-MN (*N* = 14)	*p* Value
Age, years, [median (IQR)]	61 (50, 64)	70 (54, 72)	0.63
Male, n, (%)	1 (20.0)	8 (57.1)	0.15
Serum creatine, mg/dl, [median (IQR)]	0.77 (0.56, 1.00)	0.83 (0.71, 0.88)	0.83
Serum IgG, mg/dl, [median (IQR)]	540 (271, 623)	654 (593, 1033)	0.02
Serum total protein, g/dl, [median (IQR)]	4.6 (3.6, 4.7)	5.5 (4.8, 6.3)	0.08
Urine protein, g/gcre, [median (IQR)]	7900 (5327, 8778)	2647 (1114, 4980)	0.049
Allergy disease, n, (%)	3 (60.0)	1 (7.1)	0.01
Malignancy, n, (%)	2 (40.0)	1 (7.1)	0.08

Table 2 summarizes the characteristics of five THSD7A-MN cases. Three of five patients were positive for THSD7A antibody as determined by ELISA (EUROIMMUN, Lübeck, Germany). Of these three patients, two patients had not received immunosuppressive therapy, mainly oral glucocorticoid and are in complete remission. In three patients who received immunosuppressive therapy, only one with severe asthma and eosinophilia did not experience complete remission in spite of receiving many immunosuppressive agents (oral glucocorticoid, cyclosporine and mizoribine).

**Table 2.** Characteristics of THSD7A patients with membranous nephropathy.

Case	Age	Sex	Urinary protein (g/gCr)	Serum THSD7A |antibody (ELISA)	Co-morbidity	Treatment	Prognosis
1	50	Male	12.7	Not performed	Asthma Eosinophilia	Predonisolone Cyclosporine Mizoribine	Partial remission
2	30	Female	3.2	positive	Eosinophilia	Predonisolone	Complete remission
3	61	Female	5.3	positive	Thymoma Asthma	No immunosuppresive	Complete remission
4	88	Female	8.8	Not performed	Gastric cancer Rheumatoid arthritis	Predonisolone	Complete remission
5	64	Female	9.1	positive	No	No immunosuppresive	Complete remission

In our single-center cohort study, we described and compared the characteristics of PLA2R-MN and THSD7A-MN. In our study, THSD7A-MN accounted more for primary MN compared to previous report [2]. As reported previously [3], we found malignancy (gastric cancer and thymoma) in two out of five patients (40%) in THSD7A-MN group. We also found allergic disease is more prevalent in THSD7A-MN group than PTA2R-MN group.

Hoxha et al. [3] reported that out of 25 MN patients with serum THSD7A antibodies, as many as 7 (28%) had a malignant tumor. THSD7A expression was reportedly high in colorectal and breast cancer tissues [4]. Therefore, aggressive cancer screening is advised for PLA2R-negative MN patients, particularly those positive for THSD7A. Tumor tissues obtained from THSD7A-MN patients was not necessarily positive for THSD7A [5]. PLA2R-MN has been recently reported to be associated with malignancy [5]. However, whether primary MN (PLA2R-MN and THSD7A-MN) and cancer exist just coincidentally or are pathogenetically associated is still unclear.

Only a few studies have reported the relationship between MN and allergic disease before THSD7A-MN was reported. In another Japanese study, 4 of 14 patients (28.6%) had allergic disease (1 case: eosinophilic pneumonitis) as similar to our study [2]. In our study, 2 patients had eosinophilia. In one case, both eosinophilia and proteinuria were refractory for immunosuppressive therapy [6]. In contrast, another case showed good response to glucocorticoid treatment, leading to no recurrence of eosinophilia [7]. Matsumoto et al. [8] reported that the eosinophils of patients with angiolymphoid hyperplasia with eosinophilia expressed vascular endothelial growth factor-A, which upregulated THSD7A expression, especially under Th2-prone conditions in cultured human umbilical vein endothelial cells. In fact, a significant increase in IgG4 level in the presence of IL-4 (TH2 cytokine) was observed in idiopathic MN [9]. For a clinical course and basic study, we consider that THSD7A-MN is associated with eosinophilia. However, the reason behind THSD7A expression in podocytes and not in endothelial cells in THSD7A-MN is unclear. Therefore, further investigation is required to understand the mechanism of THSD7A-MN development. Limitation of this study is small sample size, particularly THSD7A-MN.

In conclusion, in our cohort study of primary MN with THSD7A-MN or PLA2R-MN, we found that THSD7A-MN may be associated with allergic disease, especially eosinophilia as well as malignancy.
